# Objective evaluation of gait abnormalities in dogs with a thoracolumbar myelopathy using a pressure-sensing walkway

**DOI:** 10.3389/fvets.2025.1727929

**Published:** 2026-01-14

**Authors:** Léonie Straß, Sebastian Meller, Friederike Twele, Holger A. Volk

**Affiliations:** 1 Department of Small Animal Medicine and Surgery, University of Veterinary Medicine Hannover, Hannover, Germany; 2 Centre for Systems Neuroscience Hannover, Hannover, Germany

**Keywords:** ataxia, dog, gait analysis, pressure-sensing walkway, spinal cord injury

## Abstract

Objective gait assessment remains essential for evaluating ambulatory function in dogs with spinal cord injury, as subjective scales depend on training and examiner experience, limiting their sensitivity to subtle changes. A rapid, standardised, and objective method is therefore needed to assess gait abnormalities and to monitor recovery over time. This study evaluates the use of a pressure-sensing walkway to objectively quantify gait characteristics in dogs with thoracolumbar myelopathy compared to healthy controls. By analysing spatio-temporal and kinetic parameters, key differences relevant to clinical monitoring were identified. Significant differences in standard deviation values were observed between controls and dogs affected by thoracolumbar myelopathy for step/stride ratio, stance phase and swing phase. Furthermore, the coefficient of variation (CV) also differed significantly between groups for step/stride ratio, stance and swing phase. CV, a marker for ataxia, was increased not only in the pelvic but also in the thoracic limbs. Lateral skewness, thoracic limb force distribution, and pelvic limb symmetry index also differed significantly between groups, with neurologically affected dogs exhibiting higher values across all parameters. In addition, a negative correlation between the Texas Spinal Cord Injury Score and the CV was identified, indicating that the CV may contribute to an objective quantification of impairment. These findings support the use of pressure-sensing walkways as a feasible and informative modality for the standardised assessment of canine patients with thoracolumbar myelopathy.

## 1 Introduction

Intervertebral disc disease (IVDD) constitutes one of the most prevalent neurological disorders in dogs and represents a leading cause of acute onset paresis and plegia (1). A retrospective study by Rossi et al. (1) demonstrated that 72% of 845 dogs presented to small animal emergency clinics with acute non-ambulatory paresis or plegia were diagnosed with IVDD. The majority of affected dogs present with a T3-L3 myelopathy (2), reflecting the high prevalence of thoracolumbar disc disease. IVDD is a complex condition with variable clinical manifestations, ranging from isolated spinal hyperaesthesia to severe neurological deficits, including paresis, plegia, ataxia, and loss of deep pain perception (3).

Despite ongoing efforts to refine clinical scoring systems, objective assessment of gait remains a critical component in the evaluation of ambulatory function in dogs with spinal cord injury (SCI). A range of clinical gait scales has been developed, from dichotomous ambulation categories and the modified Frankel scale (MFS) to more refined ordinal measures such as the Texas Spinal Cord Injury Score (TSCIS) and the Open Field Scale (OFS). The latter 12-point scales are highly reliable and allow a finer differentiation of gait quality than simpler systems (4). However, as ordinal measures they remain limited by a ceiling effect, particularly once basic ambulation is regained, at which point subtle residual deficits are no longer consistently captured (4). Further, subjective scales require specific training and rely on the examiner’s experience and clinical background, which may limit their consistency and sensitivity in detecting subtle changes over time (4–7). This shortcoming is further compounded by the fact that the human eye cannot capture the full complexity of locomotor patterns (8, 9), resulting in poor agreement of purely observational gait analyses in dogs with thoracolumbar myelopathy, whether based on direct observation or on video recordings (10). Consequently, precise and objective quantification, especially in recovered ambulatory patients, necessitates the use of instrumented methods such as force plates, pressure-sensing walkways, instrumented treadmills, or camera-based systems. These modalities provide quantitative parameters that reveal subtle deficits and enable sensitive monitoring of recovery and treatment effects (11–13).

Kinematic gait analysis systems (2D- and 3D-systems) allow detailed evaluation of joint movements and have, for example, been applied to monitor recovery in Dachshunds with T3-L3 myelopathy after hemilaminectomy (14). However, their use is largely limited to research facilities due to high acquisition costs, complex set-up, and error susceptibility caused by marker displacement, camera positioning, or skin movement artefacts (14–17). Variability is particularly pronounced in small dogs, where short inter-marker distances amplify the impact of such errors (14).

Kinetic methods such as force plates (FP) are considered the gold standard of objective gait analysis. They capture ground reaction forces (GRF) in three orthogonal directions (vertical, craniocaudal, mediolateral) and allow the calculation of derived parameters including peak vertical force (PVF), vertical impulse (VI), centre of pressure (COP), and the moments around each axis (12). Nevertheless, their limited surface area complicates the assessment of small breeds, as simultaneous paw contacts can invalidate measurements (18, 19). Moreover, capturing consecutive footfalls in dogs of different sizes requires multiple force platforms arranged in series, which is cost-prohibitive (20). Treadmill systems with integrated multiple force plates have been developed to overcome these limitations. Sherif et al. (21) combined a four-belt force plate treadmill, ensuring separate paw strikes, with a camera-based marker system to capture objective gait parameters in dogs with thoracolumbar myelopathy. However, this method required extensive habituation and analysis, making it highly time-consuming (21) and therefore impractical for routine clinical use. Moreover, the need for prolonged habituation and a fixed treadmill speed renders this approach unsuitable for paraparetic or ataxic dogs, as many of them are only capable of walking a few consecutive steps.

Pressure-sensing walkways (PSW) represent a practical alternative. Although they do not directly measure GRFs but only vertical pressure values (PFz), calibration enables estimation of PVF and VI. PSWs additionally provide visualisation of paw contact areas, intra-paw pressure distribution, and centre of pressure, along with spatio-temporal parameters such as stance and swing phase, stride length, and velocity. Their simple handling, rapid data acquisition, and suitability for capturing consecutive strides make them particularly attractive for clinical and translational research, and most importantly can be integrated in a routine clinical workflow. Previous studies have already demonstrated their utility in distinguishing neurological cases from sound dogs as well as from those with orthopaedic gait disorders (20, 22, 23), thereby supporting their role as a feasible tool for the objective quantification of locomotor deficits in dogs with T3-L3 myelopathy. Gordon-Evans et al. (22) successfully applied PSWs to quantify gait alterations in dogs with T3-L3 myelopathy, and subsequent studies showed increased variability not only in the affected pelvic limbs but also in stride length of the thoracic limbs (20). Sherif et al. (21) confirmed increased coefficient of variation (CV) in the thoracic limbs using a force-instrumented treadmill, affecting both spatio-temporal and kinetic parameters. The CV reflects the variability of a parameter, with higher CV values indicating greater inconsistency of gait patterns and, consequently, a higher degree of ataxia (21), which is defined as impaired coordination of voluntary movement (24). These findings support the suitability of CV as a quantitative marker of clinical severity (20, 21). However, since it was previously assumed that thoracolumbar spinal cord injury primarily affects the pelvic limbs (24, 25), the observation of increased variability in thoracic limb parameters was unexpected. It remains unclear whether such results can be consistently reproduced across different systems and parameters or whether observed differences are partly attributable to methodological effects. Moreover, it is not yet known whether these parameters change in a linear fashion with increasing severity of impairment.

Thus, the aim of the present study was to investigate whether PSWs, as a simple and time-efficient method suitable for clinical routine, can reliably detect and quantify gait variability in dogs with T3-L3 myelopathy, to assess the potential of CV as an objective marker of clinical severity, and to determine whether thoracic limbs are also affected in addition to the pelvic limbs.

## 2 Methods

### 2.1 Data collection

Data were collected as part of the standardised clinical and neurological examination at the Department for Small Animal Medicine and Surgery of the University of Veterinary Medicine Hannover, Germany. Written informed consent was obtained from all owners.

Dogs in the healthy control group were required to have unremarkable orthopaedic and neurological examinations performed by a board-certified surgeon and neurologist, with no history of orthopaedic or neurological disorders or other relevant comorbidities. Dogs in the neurological group were eligible if diagnosed with a neurological disease causing a T3-L3 myelopathy and presenting with ambulatory paraparesis, while showing no abnormalities on orthopaedic examination. Dogs were excluded if they had any evidence of orthopaedic disease or a history of orthopaedic surgery. The sample size was determined with reference to comparable studies of canine SCI assessed by PSW (20, 23). A recent study provided a PASS-based power calculation indicating that approximately 15 dogs per group are required to achieve 80% statistical power (23). In alignment with this estimation, the cohort sizes in the present study were selected to reflect these reported requirements.

The PSW encompassed an 8.75-metre-long testing area. Two PSWs (Molibso, Germany) with external dimensions of 212 × 60.5 × 2.1 cm (L × W × H) were flanked at both ends by one-meter-long sensor-free extensions of identical surface material and a non-slip rubber mat, enabling unimpeded entry and exit during locomotion trials. Each platform contains 15,360 sensors, each performing 200 measurements per second. For data acquisition and analysis, the software Animal Analysis Suite version 2.10.0 (Zebris, Germany) was used. Prior to data collection, all dogs were weighed and their height at the withers was measured with the dog standing evenly on a flat surface, from the ground to the top of the withers. Subsequently, the PSW was calibrated to the weight of the subjects, which then were allowed to move freely within the setup to promote acclimatisation and reduce stress-related influences on gait patterns. Dogs were walked on a lead, with the handler remaining consistently on one side of the walkway, so each dog completed trials from both sides to reduce influence of leash side (26). Dogs were walked at a slow pace, with a minimum of three trials per direction. The walking speed was determined by the dog itself and was only manually adjusted by the handler if the dog began to transition into a trot.

In parallel, synchronised video recordings were obtained using two GoPro HERO13 Black cameras (GoPro, USA), each recording at 4 K resolution and 120 frames per second. One camera was positioned to capture both frontal and rear views, while the other provided a lateral perspective. This dual-camera setup enabled the retrospective identification of valid gait cycles during the data analysis process.

### 2.2 Data processing

The software automatically computed mean values for all parameters and standard deviations for selected variables. For analysis, data from the left and right limbs were pooled for both thoracic and pelvic limbs, respectively. Supplementary Table 1 contains the definitions of gait parameters.

Only sequences in which the dog moved in a steady and undisturbed manner were selected to minimise external influences and avoid data distortion. Initial paw placements were automatically assigned by the software and subsequently reviewed and corrected by an experienced observer in cases of misidentification.

Lastly, the CVs for standardised gait parameters were calculated to reduce the effects of difference in size, speed and body weight, thereby facilitating relative comparisons. This approach has been applied in several previous studies investigating ataxia in dogs (21, 27–29), as well as in research on horses (30) and humans (31, 32). The CV was determined as the ratio of the standard deviation (SD) to the mean. The larger the CV the more variable were the stride parameters of the dog.

### 2.3 Statistical analysis

Statistical analyses were carried out using GraphPad Prism 10 (GraphPad Software, Inc., United States). Beside conducting descriptive statistics, data were initially tested for normality using the Shapiro–Wilk test. A Gaussian distribution was rejected for all variables except for lateral and anterior–posterior (ant.-post.) skewness and force distribution.

Difference in sex distribution between groups was assessed using Fisher’s exact test. For paired data showing a normal distribution, paired *t*-tests were performed. In cases of paired non-parametric data, Wilcoxon signed-rank tests were used. For unpaired parametric data, unpaired *t*-tests with Welch’s correction were applied, whereas unpaired non-parametric data were analysed using the Mann–Whitney *U* test.

The non-parametric Spearman’s rank correlation test was applied to assess the strength and direction of non-parametric, unpaired variables.

All tests were twο-sided and a *p*-value of less than 0.05 was considered statistically significant. *p*-values were then reviewed using a False Discovery Rate (FDR) to reduce Type-I error of multiple comparisons. The two-stage step up method selected for setting FDR was designed by Benjamini, Krieger and Yekutieli (33). Values are presented as mean ± standard deviation (SD), median (range) and 95% confidence interval (CI).

## 3 Results

### 3.1 Study animals

A total of 30 dogs were included in the study, comprising 15 clinically healthy controls and 15 dogs with a T3-L3 myelopathy. An overview of group composition is presented in Table 1. No statistically significant difference in age (control: 4.43 ± 2.35; T3-L3 SCI: 5.87 ± 3.04; *p* = 0.1765) or sex distribution between the groups (*p* = 0.7152) was found. However, significant differences were observed in bodyweight (control: 18.83 ± 10.37; T3-L3 SCI: 9.81 ± 5; *p* = 0.002), with control dogs being heavier, and being taller in regard of shoulder height (control: 45.8 ± 13.76; T3-L3 SCI: 30.13 ± 5.7; *p* = 0.0007), resulting in showing higher average velocity (control: 0.91 m/s ± 0.04; T3-L3 SCI: 0.61 m/s ± 0.07; *p* < 0.0001).

**Table 1:** Group composition.

Group	Category	Control (*n* = 15)	T3-L3 SCI (*n* = 15)	Total (*n* = 30)	*p*-value
Age (years)^a^	Mean ± SD	4.43 ± 2.35	5.87 ± 3.04	5.15 ± 2.77	0.1765
	Median (range)	3.3 (2.0–10.6)	5.5 (2.0–12.3)	4.6 (2.0–12.3)	
Height (cm)^b^	Mean ± SD	45.8 ± 13.76	30.13 ± 5.7	37.97 ± 13.06	0.0007*
	Median (range)	50 (23–71)	29 (23–44)	35 (23–71)	
Back Length (cm)^b^	Mean ± SD	47.2 ± 10.51	35.33 ± 6.98	41.27 ± 10.64	0.0013*
	Median (range)	45 (33–65)	34 (25–52)	37.5 (25–65)	
Weight (kg)^a^	Mean ± SD	18.83 ± 10.37	9.81 ± 4.96	14.32 ± 9.21	0.002*
	Median (range)	17 (7–42)	7.6 (4.4–23.1)	12.25 (4.4–42.0)	
Body condition score (1–9)^a^ (66)	Mean ± SD	5.2 ± 1.01	6.07 ± 1.16	5.63 ± 1.16	0.0373*
	Median (range)	5 (4–7)	6 (5–9)	5.5 (4–9)	
Sex^c^	Male	2	6	8	0.7152
	Male-neutered	4	2	6	
	Female	8	3	11	
	Female-neutered	1	4	5	
Breed-distribution		Three Australian Sheperd, 1 Beagle, 5 Crossbreed, 2 Dachshund, 1 French Bulldog, 1 Labrador Retriever, 1 Black Russian Terrier, 1 Tibetan Terrier	One Beagle, 3 Crossbreed, 6 Dachshund, 2 French Bulldog, 1 Havanese, 1 Yorkshire Terrier, 1 Cavalier King Charles Spaniel	Three Australian Sheperd, 2 Beagle, 8 Crossbreed, 8 Dachshund, 3 French Bulldog, 1 Labrador Retriever, 1 Black Russian Terrier, 1 Tibetan Terrier, 1 Havanese, 1 Yorkshire Terrier, 1 Cavalier King Charles Spaniel	

Superscript letters indicate which statistical test was applied to compare groups (a: Mann–Whitney *U* test; b: unpaired *t*-test with Welch’s correction; c: Fisher’s exact test). A *p*-value < 0.05 (*p < 0.05) was considered statistically significant.

In the neurologically affected population, only neurologically affected dogs capable of walking independently were included and graded based on the Texas Spinal Cord Injury Score (TSCIS), with a median score of 35 (32–39) (Supplementary Table 3). In addition to paraparesis, the neurological examination also revealed proprioceptive ataxia in 12 dogs. Thirteen dogs with T3-L3 myelopathy underwent magnetic resonance imaging (MRI), which confirmed spinal cord injury secondary to intervertebral disc extrusion (IVDE); five dogs were assessed with the PSW pre-operatively, while eight were assessed post-surgery. In two cases, the neuroanatomical localisation to the T3-L3 spinal cord segments was established clinically, but this could not be confirmed due to the absence of MRI diagnostics. Both of these dogs were managed conservatively.

### 3.2 Spatio-temporal gait parameters

**Figure 1:**
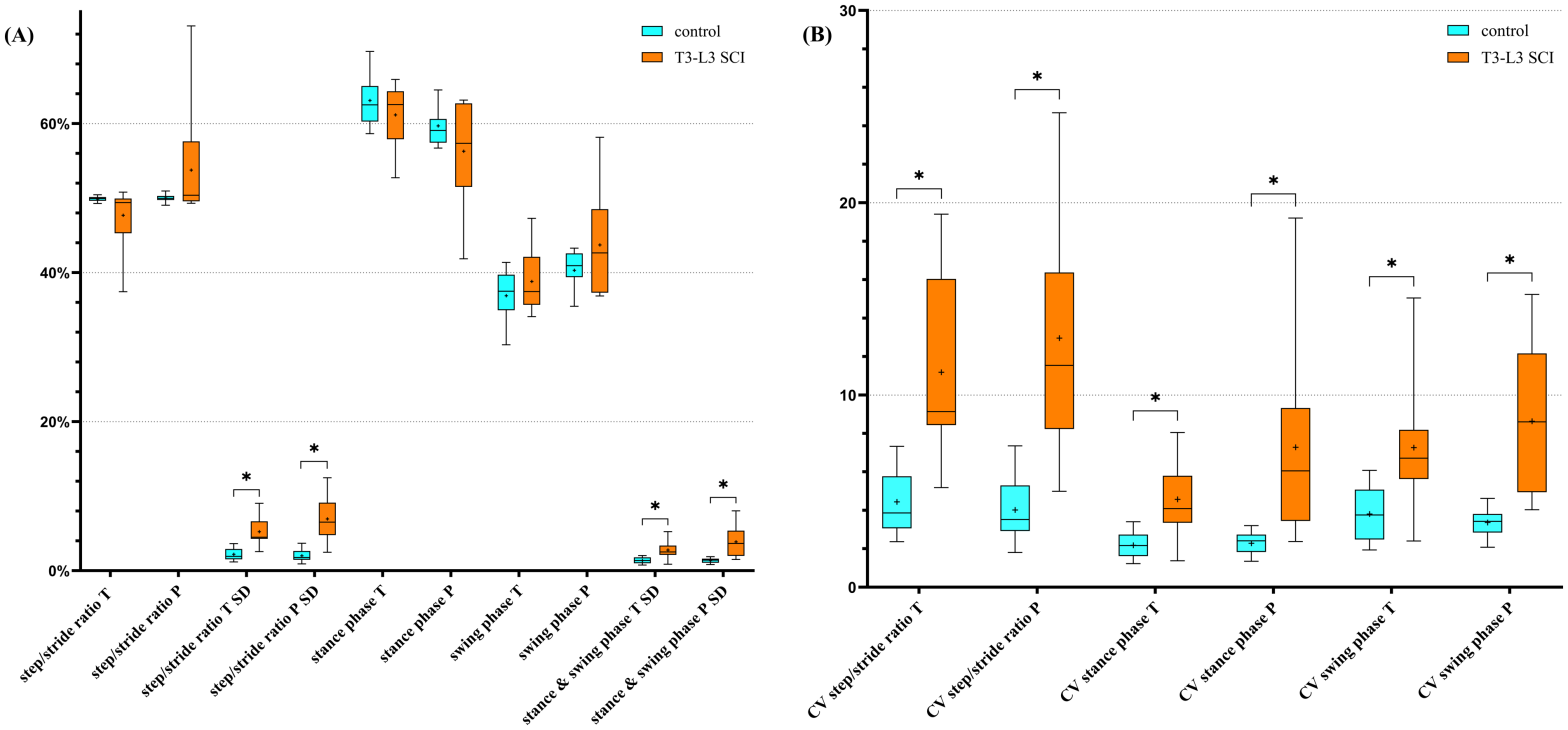
Box and whiskers plots showing comparison of spatio-temporal parameter values as **(A)** percentage and **(B)** coefficient of variation (unitless) (*p < 0.05, FDR *Q* = 0.05) between controls and dogs with a T3-L3 myelopathy. The boxes illustrate the 25–75% interquartile range with the median as a black horizontal line. The whiskers illustrate the minimum and maximum. The cross indicates the mean. T, thoracic limbs; P, pelvic limbs; SD, standard deviation; CV, coefficient of variation; SCI, spinal cord injury. See Supplementary Table 1 for detailed definitions of parameters and Supplementary Table 2 for descriptive statistics.

Significant differences in SD values for step/stride ratio, stance phase and swing phase, with dogs affected by T3-L3 myelopathy exhibiting higher values compared to the control group for both the thoracic and pelvic limbs, are highlighted in Figure 1 (additional information can be found in Supplementary Table 4). A similar pattern was observed for the coefficient of variation of step/stride ratio, stance and swing phase, which was also increased for the group with SCI (Figure 1).

### 3.3 Kinetic gait parameters

**Figure 2:**
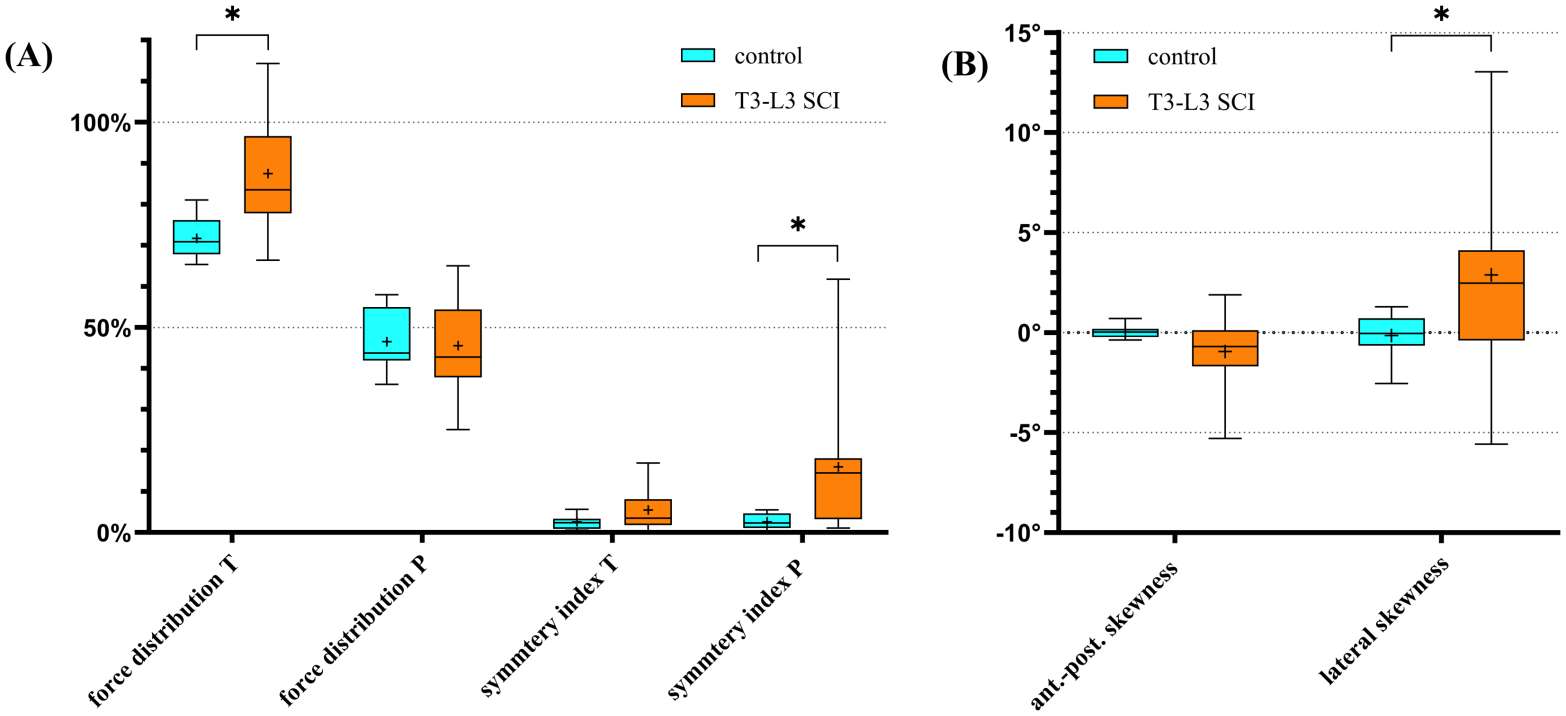
Box and whiskers plots showing the comparison of kinetic parameter values between controls and dogs with a T3-L3 myelopathy as **(A)** percentage and **(B)** degree (*p < 0.05, FDR *Q* = 0.05). The boxes illustrate the 25–75% interquartile with the median as a black horizontal line. The whiskers illustrate the minimum and maximum. The cross indicates the mean. T, thoracic limbs; P, pelvic limbs; ant.-post., anterior–posterior; SCI, spinal cord injury. See Supplementary Table 1 for detailed definitions of parameters and Supplementary Table 2 for descriptive statistics.

Supplementary Table 5 and Figure 2 illustrate that, beyond differences in lateral skewness, force distribution in the thoracic limbs, and symmetry index of the pelvic limbs also showed significant group-related differences, with dogs affected by T3-L3 SCI showing higher values.

### 3.4 Correlation between symmetry index, coefficient of variation, and Texas spinal cord injury score

The analysis revealed significant negative correlations of moderate to strong magnitude between the CV of stance phase P (*r*: −0.7197; CI: −0.9032 to −0.3136; *p* = 0.0036), swing phase P (*r*: −0.6917; CI: −0.8924 to −0.2623; *p* = 0.0058), step/stride ratio P (*r*: −0.5664; CI: −0.8411 to −0.0596; *p* = 0.0303) and TSCIS, whereas the remaining associations were not statistically significant (Table 2).

**Table 2:** Correlation between coefficient of variation, symmetry index and Texas spinal cord injury score.

Parameter	*r*	CI	*p*
CV stance phase P	−0.7197	−0.9032 to −0.3136	0.0036*
CV swing phase P	−0.6917	−0.8924 to −0.2623	0.0058*
CV step/stride ratio P	−0.5664	−0.8411 to −0.0596	0.0303*
Symmetry index P	0.2841	−0.2824 to 0.7038	0.3014
CV stance phase T	−0.2411	−0.6797 to 0.3244	0.3826
CV swing phase T	−0.1338	−0.6151 to 0.4202	0.6302
CV step/stride ratio T	0.02617	−0.5053 to 0.5432	0.9276

*r* = Spearman’s rank correlation coefficient; CI = 95% confidence interval; *p < 0.05.

T, thoracic limbs; P, pelvic limbs; SD, standard deviation; CV, coefficient of variation.

Spearman’s rank correlation was used to assess the strength and direction of unpaired non-parametric variables.

See Supplementary Table 1 for detailed definitions of parameters and Supplementary Table 2 for descriptive statistics.

## 4 Discussion

In this study, we tested whether a PSW provides reliable, clinically meaningful metrics to quantify locomotor impairment in dogs with T3-L3 SCI, complementing current subjective scoring systems. Compared with controls, dogs with T3-L3 SCI exhibited larger SDs and CVs across spatiotemporal gait variables, indicating that increased step-to-step variability is a key feature of their gait disturbance. Consistent with this, higher CVs for step/stride length, swing, and stance phase were moderately to strongly negatively correlated with TSCIS, i.e., variability increased as clinical function worsened. Additional PSW-derived measures—greater lateral deviation, higher pelvic-limb symmetry indices, and a cranial redistribution of load toward the thoracic limbs—further demonstrate the sensitivity of PSWs to detect subtle yet clinically relevant alterations. Notably, CVs were also elevated in the thoracic limbs, providing objective evidence that thoracic limb gait is secondarily affected in addition to pelvic-limb deficits, in agreement with prior work using different quantitative gait tools (20, 21). Together, these findings challenge the traditional view that thoracolumbar lesions exclusively impair pelvic-limb locomotion and underscore the clinical relevance of assessing both thoracic and pelvic limbs with quantitative gait analysis.

Comparison with previous studies highlights both consistencies and distinct deviations in the characterisation of gait alterations associated with thoracolumbar SCI. In the context of vertical force distribution, it should be noted that previous studies in neurologically impaired dogs have reported partly divergent results. In the present cohort, dogs compensated in accordance with the observations of Gordon-Evans et al. (22), exhibiting higher vertical PVFs in the thoracic limbs, most likely reflecting a cranial shift of body weight to compensate for the paraparesis and ataxia in the pelvic limbs. This interpretation is supported by findings on the COP, which was located more cranially in dogs with SCI compared with neurologically unremarkable individuals (34). Such a cranial displacement of the COP is presumed to represent a compensatory forward loading strategy in response to chronic pelvic limb paresis (34).

In contrast, Park et al. (23) reported no significant differences in PVFs between affected and control dogs, with no differences detected in either the thoracic or the pelvic limbs. Similarly, another study found no significant differences in PVFs between the pelvic limbs of neurologically normal Dachshunds and those that had undergone hemilaminectomy for thoracolumbar disc disease (14). Within the postoperative group, however, the limbs that had been more severely affected preoperatively exhibited significantly higher PVF values (14).

These contradictory results may be explained by differences in study populations. Breed-specific conformational characteristics of trunk and limbs are considered a major source of variability in PVFs (35). For example, Dachshunds accounted for 70% of the dogs in the study by Gordon-Evans et al. (22) and 40% of the present cohort and were therefore overrepresented compared with other investigations. Morphological differences directly influence kinetics as dogs with short limbs must generate higher limb frequencies to maintain a given trunk velocity than dogs with longer limbs (35–37), resulting in breed-dependent differences in ground reaction forces (12).

Although normalisation to body weight is routinely applied to minimise the influence of size and mass and has proven a reliable approach for kinetic parameters, several studies suggest that significant breed-related differences may persist despite normalisation, particularly regarding the magnitude and distribution of GRFs (38, 39). This also applies to PVF, as it represents the maximum of the vertical GRF. Moreover, even within morphologically homogeneous populations, trial-to-trial variability in limb velocity may occur, which can substantially affect GRFs and thus PVFs (40).

Supporting this, previous studies demonstrated a negative correlation between body weight and PVF and a positive correlation between body weight and vertical impulse during walking (41). These relationships appear to be driven less by body weight per se than by differences in relative speed. Smaller dogs move relatively faster at the same absolute velocity compared with larger dogs, resulting in higher PVF values (41) as a consequence of a more dynamic paw strike (35).

The analysis of SI, which in the present study was based on PVF and served as a measure of the evenness of load distribution between paired thoracic and pelvic limbs (36), likewise revealed clear differences. It should be noted that different formulae have been applied in the literature to calculate SI, all of which derive from the concepts originally proposed by Budsberg et al. (36), but have since been modified. Depending on the method employed, different ranges and interpretations of SI values can result, which limits comparability between studies. In the present investigation, a modified formula according to Budsberg et al. (36) was applied, in which the difference in PVF between the more and less loaded limb of a pair (either left vs. right thoracic limb or left vs. right pelvic limb) is expressed relative to their sum (Supplementary Table 1). This normalisation relates the asymmetry to the total load on the pair of limbs and thereby facilitates comparison between dogs of different sizes and body weights.

The data of the present study showed clear asymmetries in the pelvic limbs as well as a lateral displacement of the centre of mass. These results suggest deficits in lateral limb control, which have previously been described in dogs with thoracolumbar spinal cord injury (27). In ambulatory dogs following spinal cord injury, a significantly increased variability (CV) of lateral paw placement of the pelvic limbs has been documented compared with healthy controls (27). Taken together, these results provide objective evidence that lateral instability constitutes a clinically relevant component of ataxia.

With regard to stance phase, the literature likewise presents a heterogeneous picture. Earlier studies described a prolonged stance phase in the thoracic limbs, interpreted as a compensatory mechanism to improve postural stability (14, 18). In the present study, however, no systematic prolongation of stance phase was observed in paraparetic dogs compared with healthy controls. Instead, significantly increased variability was detected, indicating irregularities rather than a consistent extension. Previous investigations have demonstrated a marked negative correlation between trunk velocity and stance phase duration, meaning that faster locomotion is associated with shorter ground contact times (38, 42). Contrary to this expectation, the paraparetic dogs in the present study, despite lower mean velocities, did not exhibit a prolonged stance phase. The mean velocity of the control group was 0.91 m/s, whereas the paraparetic group showed an even lower mean velocity of 0.61 m/s. By comparison, higher velocities were reported in other studies. Dogs in the study of Sherif et al. (21) walked on a treadmill at a maximum speed of 0.7 m/s, Gordon-Evans et al. (22) reported mean velocities of 1.0 m/s (range: 0.45–1.45 m/s) in healthy and 0.79 m/s (range: 0.36–1.39 m/s) in affected dogs, while Park et al. (23) performed gait analysis at controlled velocities of 0.8–1.4 m/s. The prolongation of stance phase in the thoracic limbs observed by Park et al. (23) may also explain why the PVFs measured in that study were lower than those in the present investigation. Budsberg et al. (41) had already demonstrated that a prolongation of stance phase is accompanied by a decrease in PVF.

Overall, these findings indicate that slower absolute velocity does not necessarily coincide with a prolonged stance phase. Rather, as previously outlined, it must be considered that smaller dogs move relatively faster than larger dogs at the same absolute velocity. This may result in shortened stride and stance times, as demonstrated in another study in which affected dogs developed increased thoracic limb velocity as a compensatory mechanism (22). Comparability between studies therefore remains limited, as both velocity and methodological conditions varied (12, 19). Future investigations should therefore take into account not only absolute velocity but also relative velocity and limb dynamics in order to refine the interpretation of stance duration and its pathophysiological significance.

Given the influence of velocity on kinetic and kinematic outcomes (43, 44), many gait analysis protocols aim to control this factor by defining a fixed velocity interval prior to data collection. Trials are repeated until a sufficient number of valid passes within this interval has been obtained (12). This approach is intended to minimise velocity-related variability and thereby improve the reliability and comparability of parameters across studies (11, 12). In relatively homogeneous populations of clinically healthy dogs, narrow velocity ranges (± 0.3 m/s) combined with controlled acceleration (± 0.5 m/s^2^) have proven suitable to effectively reduce data variance (35, 44). Accordingly, fixed velocity ranges between 1.0 and 1.3 m/s are frequently chosen for walking trials (45).

Despite these methodological advantages, enforcing a fixed velocity is associated with distinct limitations in heterogeneous populations or in dogs with locomotor impairment (12, 41, 45–49). Dogs with different morphometric characteristics may be forced to walk or trot outside their preferred velocity (12), which alters natural gait mechanics and can affect the parameters under investigation. In studies involving lame dogs, the use of fixed velocities was further associated with an increased number of invalid trials (45, 47–49). This required additional repetitions and simultaneously increased the risk of fatigue, which in turn may exacerbate lameness during data collection (46). Comparable challenges can also be expected in neurologically affected dogs, for whom gait impairment may make it difficult or impossible to maintain a prescribed velocity. For these reasons, the present study adopted a preferred velocity approach, allowing dogs to move at a self-selected speed within a broader velocity range.

As CVs mitigate the influence of differences in body size, walking speed, and body weight, thereby enabling relative comparisons, this further underscores their usefulness in clinical practice, particularly in heterogeneous populations. This approach can be especially valuable in cases where distinguishing between orthopaedic and neurological gait abnormalities is challenging, and reference values for different breeds at varying speeds are not yet available.

Consistent with the observations of Sherif et al. (21) the present analysis revealed increased variability in spatio-temporal gait parameters, evident through elevated CV in both thoracic and pelvic limbs. This reinforces the notion that not only the pelvic limbs but also the thoracic limbs exhibit irregularities. Rather than representing ataxia alone, this variability may also indicate compensatory adaptations aimed at maintaining balance and locomotor stability, that may not be readily perceptible to the human eye. An alternative hypothesis suggests that a sensory system may be involved which, beyond affecting the caudal segments of the injured spinal cord, also alters the coordination of thoracic limb movements (50). The generation and coordination of rhythmic limb movements during locomotion are governed by neuronal networks known as central pattern generators (CPGs). These networks are capable of producing cyclic motor output even in the absence of peripheral sensory input or supraspinal control (51). CPGs are anatomically distributed within the spinal cord, with cervical and lumbar locomotor circuits working in close coordination. Propriospinal interneurons (PINs) serve as a crucial link between these segmental CPGs by establishing ascending, descending, and crossed connections, thereby facilitating interlimb coordination (50, 52). The structural and functional organisation of the propriospinal network indicates a crucial role in synchronizing cervical and lumbar central pattern generators (CPGs), thereby mediating interlimb synchronisation during locomotion. Long propriospinal interneurons (PINs) have been shown to synchronise thoracic and pelvic limbs activity during stepping (53, 54). Disruption of long ascending PINs may impair interlimb coordination (55), and interruption of signal transmission within the thoracic spinal cord decouples rhythmic activity between the cervical and lumbar enlargements (56, 57).

In cases of T3-L3 myelopathy, desynchronisation occurs, commonly resulting in a lower step frequency of the pelvic limbs compared to the thoracic limbs. As neurological function recovers, these frequencies tend to re-align, with unidirectional input being transmitted from the lumbar CPGs to the thoracic CPGs, but not vice versa (50). This process has been utilised in clinical scoring systems to evaluate gait improvement (7). Comparable mechanisms appear to persist in humans, where propriospinal interneurons play a key role in mediating neural coupling between the cervical and lumbar spinal segments to coordinate arm and leg movements (58, 59). Further, experimental evidence from animal models indicates that propriospinal interneurons are instrumental in promoting functional recovery after spinal cord injury by forming relay pathways or detours within the damaged cord, suggesting that they may also play a significant role in motor rehabilitation in humans (60–63). The present findings underscore the clinical importance of assessing both thoracic and pelvic limb motor control in dogs and humans with spinal cord injury. Despite anatomical and functional differences between species (52), dogs with naturally occurring SCI provide a valuable translational model for investigating complex pathomechanisms under clinically relevant conditions, thereby contributing meaningfully to human spinal cord research (64, 65). Conversely, insights gained from human neurorehabilitation and experimental studies can inform veterinary therapeutic approaches. This bidirectional perspective enhances our understanding of spinal cord pathology and recovery and may ultimately support the development of more accurate prognostic indicators and more effective, targeted rehabilitation protocols across species.

The negative correlation between the CV for pelvic limb parameters and the TSCIS further highlights the potential of using CV as an objective marker to quantify the severity of neurological impairment. In this context, higher CV values correspond to greater variability of gait parameters and consequently, to a higher degree of ataxia (21), underscoring the suitability of CV as a quantitative measure of clinical severity. To fully explore its clinical relevance, future studies should investigate the longitudinal development of CV during the course of recovery and assess its association with neurological status. Moreover, evaluating CV in relation to long-term functional outcome could help determine its prognostic value and clarify whether specific patterns of variability are indicative of recovery potential.

Accurate automatic assignment of paw contacts on the pressure-sensing walkway proved particularly challenging in neurologically affected dogs, making data evaluation highly time-consuming. Despite the increased availability of raw data, unfiltered recordings differed considerably from manually processed datasets, which emphasises the current necessity of extensive manual data processing and thereby limits immediate clinical implementation. Nevertheless, pressure-sensitive walkways remain highly useful as they require no prolonged acclimatisation period and are therefore suitable for neurologically impaired animals. They further enable the evaluation of several consecutive steps and provide both spatio-temporal and kinetic parameters without the need for complex or time-consuming setups, while being applicable across a wide range of dog sizes. Most importantly, they generate objective quantitative data capable of detecting even subtle gait alterations, as demonstrated in the present study.

## 5 Limitations

Methodological and practical limitations must be considered when interpreting the findings of this study. Despite the promising diagnostic potential, the study is limited by a small and breed-biased sample, with an overrepresentation of chondrodystrophic dogs such as Dachshunds. While this reflects the clinical population, larger and more heterogeneous cohorts, including dogs with lesions at different spinal levels, are necessary to validate and generalise the findings. Furthermore, longitudinal assessments and breed-matched case-control comparisons would further strengthen the interpretability of the results. In two cases, owners declined further diagnostic imaging via MRI, meaning that a T3-L3 myelopathy due to IVDD could only be presumed based on clinical signs. In addition, the presence of intravenous catheters in some dogs, as well as the use of pain medications, must also be considered, since both may influence gait patterns. Moreover, as gait speed was not standardised to a fixed velocity interval, this may have introduced velocity-related variability in kinetic and spatio-temporal parameters. Consequently, future studies should therefore consider the use of relative velocity metrics to minimise speed-related confounding and improve comparability between subjects. It should also be noted that, despite a negative history, thorough clinical examination, and unremarkable MRI findings in the T3-L3 region, the presence of orthopaedic problems cannot be completely ruled out in either control or neurologically affected dogs. Finally, automatic assignment of paw contacts on the PSW was particularly challenging in neurologically affected dogs, which necessitated extensive manual processing of the data and currently limits immediate clinical implementation. Overall, general divergences may result from differences in lesion severity, chronicity, or underlying compensatory strategies employed by the animals, and demands further research.

## 6 Conclusion

This study demonstrates that objective gait analysis using a PSW can detect subtle locomotor abnormalities in dogs with neurological deficits due to T3-L3 SCI. The neurological group exhibited significant changes in spatiotemporal parameters, including SD and CV in both thoracic and pelvic limbs, suggesting increased variability across all limbs. These findings may challenge current assumptions regarding compensatory mechanisms in T3-L3 SCI, indicating a more global gait disturbance than previously assumed. Kinetic analysis further revealed significant group-related differences in lateral skewness, thoracic limb force distribution, and pelvic limb symmetry index, with higher values observed in dogs with T3-L3 myelopathy. Notably, the observed negative correlation between CV and the TSCIS score supports the potential of CV as a sensitive, objective parameter for detecting subtle motor deficits and for monitoring disease progression over time. These results suggest that CV may serve as a valuable complement to clinical scoring systems in both diagnostics and therapeutic follow-up.

## Data Availability

The original contributions presented in the study are included in the article/Supplementary material, further inquiries can be directed to the corresponding author.
