# Supplementary material for: Objective evaluation of gait abnormalities in dogs with a thoracolumbar myelopathy using a pressure-sensing walkway
Source: Front Vet Sci. 2026 Jan 14;12:1727929. doi: 10.3389/fvets.2025.1727929 (PMC12849788; doi:10.3389/fvets.2025.1727929)
Supplement: Supplementary file 1 [file Data_Sheet_1.pdf]

Supplementary Material

1 Supplementary Figures and Tables

Supplementary Table 1: Modified definitions of gait parameters by Zebris Medical GmbH (2023). Animal Analysis Suite: Software Manual (Version 2.8.x.) [PDF].

**Parameter**	**Unit**	**Definition**
**Spatio-temporal**		
Stride length	cm	The stride length is the distance covered by the dog's trunk between two consecutive touchdowns of the same limb. It is measured as the distance between the pressure plot of one paw to the consecutive pressure plot of the same paw. There is only one value for the stride length, and on average over several steps, it does not depend on which paw it is measured from.
Step length	cm	The step length for the four limbs is defined as follows:<br>Left hind is the distance from right hind to left hind contacts.<br>Right hind is the distance from left hind to right hind contacts.<br>Left front is the distance from right front to left front contacts.<br>Right front is the distance from left front to right front contacts.
Stance phase	%	Period within a gait cycle during which one limb is in contact with the ground (stemming phase).
Swing phase	%	Period without ground contact within a gait cycle.
Step/stride ratio	%	Step to stride ratio is the step length of each leg divided by the stride length (same for all legs) and multiplied to 100%.
Velocity	m/s	Average distance travelled per second.
**Kinetic**		
Force distribution	%	The maximum force in % of the body weight determined from the contacts in the analysis interval for each paw.<br>$Force\ distribution = \frac{Peak\ vertical\ force\ [N]}{(Body\ weight\ \ [kg]\ x\ 9.81)} x100$<br>9.81m/s^2 = Standard gravitational acceleration
lateral skewness;<br><br>anterior-posterior skewness	°	The indicated angles (skewness) result from the connection of the centres of pressure in lateral and anterior-posterior direction. The value is calculated from the skewness of the lines in relation to the vertical or horizontal line with clockwise positive rotation.
SI	%	symmetry index; Describes the symmetry of front- and hindlimb pairs and is calculated from the superior (X_s) and inferior (X_i) peak vertical force of the two limbs.<br>Modified formula by Budsberg et al. (1993) (1): $SI = \frac{(Xs-Xi)}{(Xs+Xi)} x\ 200$
PFz	N	Peak force is defined as the peak force of the average force curve.

Supplementary Table 2: Descriptive statistics.

PARAMETER	control group (n = 15)				T3-L3 SCI (n = 15)			
	MEAN	MEDIAN	MINIMUM	MAXIMUM	MEAN	MEDIAN	MINIMUM	MAXIMUM
spatio-temporal								
step/stride ratio T	49.87	49.93	49.28	50.45	47.7	49.4	37.42	50.8
step/stride ratio T SD	2.216	1.916	1.180	3.629	5.235	4.492	2.571	9.052
step/stride ratio P	50.02	49.96	49.03	50.95	53.76	50.38	49.3	73.09
step/stride ratio P SD	2.011	1.779	0.9045	3.681	6.958	6.511	2.472	12.47
stance phase T	63.1	62.51	58.63	69.68	61.17	62.55	52.73	65.91
stance phase T SD	1.383	1.378	0.7525	2.042	2.789	2.523	0.8745	5.243
stance phase P	59.69	59.08	56.71	64.51	56.29	57.35	41.85	63.14
stance phase P SD	1.363	1.413	0.8185	1.890	3.881	3.669	1.495	8.037
swing phase T	36.9	37.49	30.32	41.37	38.83	37.45	34.09	47.27
swing phase T SD	1.383	1.378	0.7525	2.042	2.789	2.523	0.8745	5.243
swing phase P	40.31	40.92	35.49	43.29	43.71	42.65	36.86	58.15
swing phase P SD	1.363	1.413	0.8185	1.890	3.881	3.669	1.495	8.037
stride length	63.04	65.04	33.59	97.42	33.02	32.73	25.38	44.25
stride length SD	1.947	1.93	0.582	3.756	3.928	2.849	1.335	11.26
step length T	31.51	32.5	16.66	48.76	15.73	15.39	11.08	20.37
step length T SD	1.336	1.221	0.608	2.313	1.753	1.409	0.9945	3.613
step length P	31.53	32.59	16.75	48.27	17.77	17.11	12.63	28.1
step length P SD	1.317	1.226	0.4835	2.628	2.311	1.941	0.9475	4.61
velocity	0.92	0.9	0.599	1.286	0.601	0.600	0.408	0.774
CV step/stride ratio T	4.446	3.86	2.370	7.330	11.19	9.14	5.180	19.400
CV step/stride ratio P	4.021	3.52	1.810	7.350	12.96	11.54	4.990	24.680
CV stance phase T	2.187	2.17	1.230	3.410	4.579	4.09	1.380	8.050
CV stance phase P	2.294	2.41	1.350	3.200	7.279	6.05	2.380	19.200
CV swing phase T	3.813	3.76	1.940	6.080	7.264	6.71	2.400	15.040
CV swing phase P	3.373	3.42	2.080	4.620	8.632	8.6	4.030	15.230
kinetic								
lateral skewness	-0.1494	-0.033	-2.542	1.304	2.887	2.484	-5.581	13.03
ant.-post. skewness	0.03827	0.036	-3.710	0.713	-0.9504	-0.704	-5.292	1.896
force distribution T	71.71	70.85	65.35	81.02	87.49	83.52	66.33	114.3
force distribution P	46.52	43.72	36.11	57.95	45.52	42.74	25.05	64.99
symmetry index T	2.612	2.396	0.541	5.645	5.529	3.521	0.427	16.94
symmetry index P	2.645	2.332	0.419	5.513	16.01	14.53	1.069	61.7

T= thoracic limbs; P= pelvic limbs; SD= standard deviation; CV= coefficient of variation
See **Supplementary Table 1** for detailed definitions of parameter.

Supplementary Table 3: Texas Spinal Cord Injury Score (TSCIS) for dogs (2).

Gait[a]	
0	no voluntary movement seen when supported
1	intact limb protraction with no ground clearance
2	intact limb protraction with inconsistent ground clearance
3	intact limb protraction with consistent ground clearance (> 75%)
4	ambulatory, consistent ground clearance with moderate paresis–ataxia (will fall occasionally)
5	ambulatory, consistent ground clearance with mild paresis–ataxia (does not fall, even on slick
6	normal gait
Proprioceptive positioning[b]	
0	absent response
1	delayed response
2	normal response
Nociception[c]	
0	no deep nociception
1	intact deep nociception, no surficial nociception
2	nociception present

[a] Ground clearance refers to the ability to lift the limb off of the ground when it is being protracted.
[b] Proprioceptive positioning is performed by supporting the dog's weight and gently placing the dorsum of the paw on the ground. A delayed response is indicated by a greater than 2s lag between paw placement and correction.
[c] Deep nociception is measured by cross-clamping the distal limb or nail bed with haemostats. Superficial nociception is tested by pinching the inter-digital webbing with haemostats.

Supplementary Table 4: Statistical comparison of spatio-temporal parameter values (*p < 0.05, FDR Q = 0.05) between controls and dogs with a T3-L3 myelopathy.

PARAMETER	control vs. T3-L3 SCI group	
	p-value	FDR threshold
step/stride ratio T	0.067496	0.141742
step/stride ratio T SD	<0.0001*	0.000001
step/stride ratio P	0.389233	0.408695
step/stride ratio P SD	<0.0001*	<0.000001
stance phase T	0.389233	0.408695
stance phase T SD	<0.0001*	0.000094
stance phase P	0.136974	0.287646
stance phase P SD	<0.0001*	0,000004
swing phase T	0.389233	0.408695
swing phase T SD	<0.0001*	0.000094
swing phase P	0.136974	0.287646
swing phase P SD	<0.0001*	0.000004
CV step/stride ratio T	<0.0001*	<0.000001
CV step/stride ratio P	<0.0001*	<0.000001
CV stance phase T	<0.0001*	0.000059
CV stance phase P	<0.0001*	0.000017
CV swing phase T	0.000253*	0.000265
CV swing phase P	<0.0001*	<0.000001

T = thoracic limbs; P = pelvic limbs; SD = standard deviation; CV = coefficient of variation.
As all data were unpaired and non-parametric, analyses were performed using the Mann–Whitney U test.
See **Supplementary Table 1** for detailed definitions of parameters and **Supplementary Table 2** for descriptive statistics**.**

Supplementary Table 5: Statistical comparison of kinetic parameter values (*p < 0.05, FDR Q = 0.05) between controls and dogs with a T3-L3 myelopathy.

PARAMETER	control vs. T3-L3 SCI group	
	p-value	FDR threshold
lateral skewness	0.029394*	0.040317
ant.-post. skewness	0.040297	0.040317
force distribution T	0.000761*	0.000799
force distribution P	0.775552	0.407165
symmetry index T	0.074204	0.038957
symmetry index P	0.001187*	0.001246

T = thoracic limbs; P = pelvic limbs; SD = standard deviation; CV = coefficient of variation.
For unpaired parametric data (skewness, force distribution), unpaired t-tests with Welch's correction were applied, whereas all remaining unpaired non-parametric data were analysed using the Mann–Whitney U test.
See **Supplementary Table 1** for detailed definitions of parameters and **Supplementary Table 2** for descriptive statistics**.**

References

1. Budsberg SC, Jevens DJ, Brown J, Foutz TL, DeCamp CE, Reece L. Evaluation of limb symmetry indices, using ground reaction forces in healthy dogs. American Journal of Veterinary Research. 1993;54(10):1569-74.

2. Levine GJ, Levine JM, Budke CM, Kerwin SC, Au J, Vinayak A, et al. Description and repeatability of a newly developed spinal cord injury scale for dogs. Preventive Veterinary Medicine. 2009;89(1):121-7.
